# Systematic review: Resting state functional MRI as a biomarker for non-invasive brain stimulation in upper limb recovery post-stroke

**DOI:** 10.1007/s10072-025-08224-5

**Published:** 2025-06-02

**Authors:** Mudasar Aziz, Sheharyar Baig, Wen Hai, Ali Ali, Arshad Majid, Li Su

**Affiliations:** 1 Division of Neuroscience, Sheffield Institute for Translational Neuroscience, The University of Sheffield, 385 A Glossop Road, Sheffield, S10 2HQ UK; 2 Department of Psychiatry, University of Cambridge, Cambridge, UK

**Keywords:** Stroke rehabilitation, Non-invasive brain stimulation (NIBS), Transcranial magnetic stimulation (TMS), Functional connectivity, Resting-state fMRI (rs-fMRI), Arm weakness recovery

## Abstract

**Background:**

Stroke is a leading cause of adult-onset disability. Non-invasive brain stimulation (NIBS) techniques such as transcranial magnetic stimulation (TMS), transcranial direct current stimulation (tDCS), and transcutaneous vagus nerve stimulation (tVNS) may improve arm weakness after stroke. Resting-state functional MRI (rs-fMRI) and near-infrared spectroscopy (rs-fNIRS) assess brain connectivity. Identifying the effect of NIBS on rs-fMRI/rs-fNIRS may illuminate the post-stroke recovery process. This systematic review assesses NIBS effects on clinical and rs-fMRI/rs-fNIRS outcomes in stroke survivors with arm weakness.

**Methods:**

Systematic searches were conducted in EMBASE and MEDLINE. Articles involving adults with arm weakness from stroke, treated with more than one session of NIBS (TMS/tDCS/tVNS) and reporting clinical and rs-fMRI/rs-fNIRS outcomes at baseline and post-intervention were included. The Cochrane Risk of Bias tool was used to assess the methodological quality of included studies. Data extraction and narrative synthesis were performed.

**Results:**

Twelve articles containing 393 participants were included. Nine studies assessed TMS, two studies assessed tDCS, and one study used dual-mode stimulation (TMS and tDCS). All studies showed significant improvements in clinical measures of arm function compared to baseline following NIBS. All studies showed changes in functional connectivity post-intervention. Enhanced interhemispheric connectivity, particularly between primary motor cortices, was positively correlated with functional outcomes.

**Discussion:**

Both TMS and tDCS are promising adjunctive therapies for arm weakness post-stroke. Rs-fMRI, particularly interhemispheric connectivity, may provide a valid biomarker of restitution of function with NIBS. Future research should involve.

## Introduction

Stroke is a major cause of long-term disability worldwide [1]. Arm weakness after stroke affects approximately 50% of stroke survivors and significantly impacts quality of life [2]. While conventional rehabilitation can improve arm function in subacute and chronic stroke [3], the intensity of rehabilitation required is costly and time-consuming to deliver at scale [4]. There is an unmet need for adjuncts that optimize or potentiate the effect of rehabilitation.

**Fig. 1:**
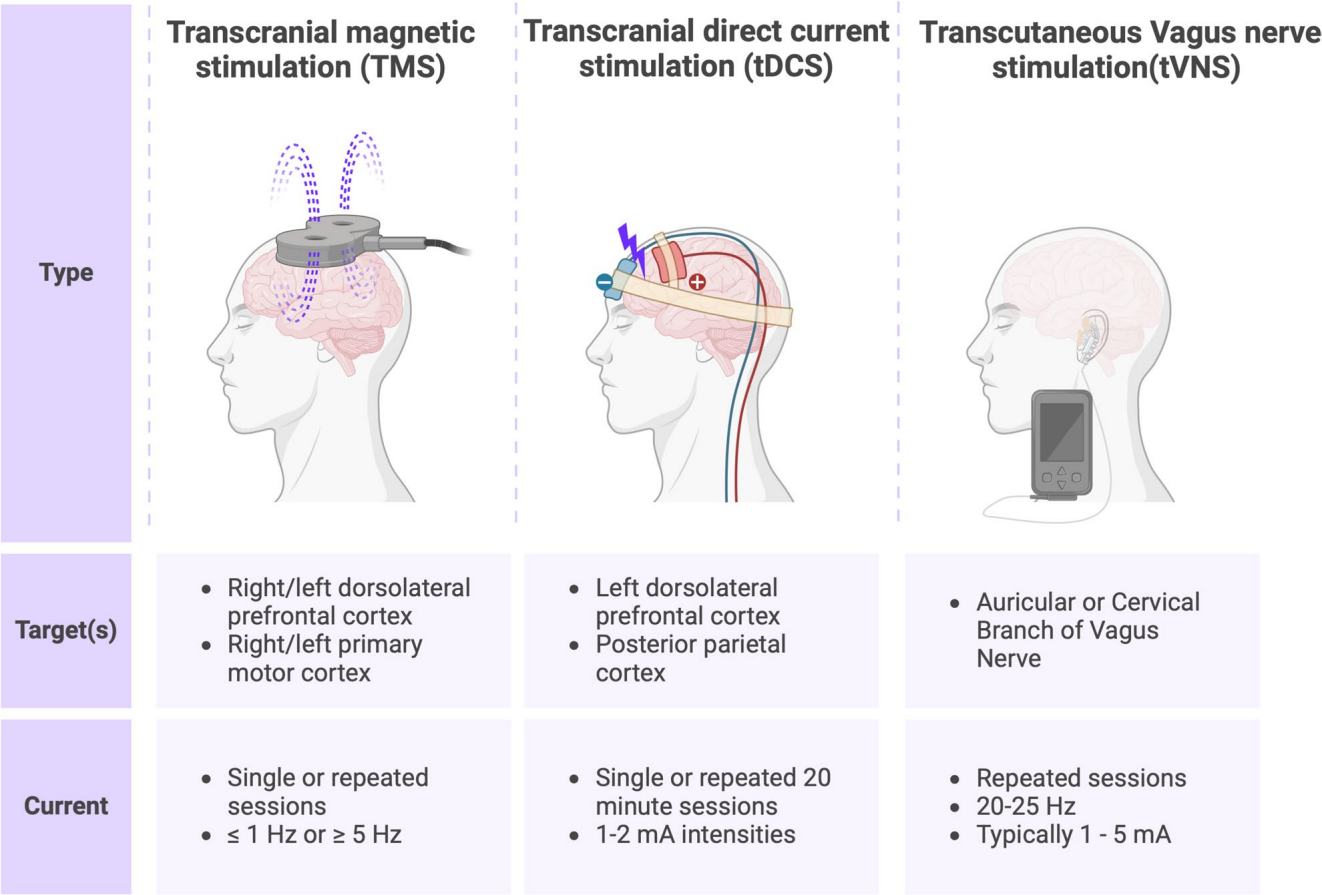
Outline of types of non-invasive brain stimulation used in studies of stroke. Created with biorender.com

Neuromodulation therapies have gradually emerged as tools through which stroke rehabilitation might be improved [5]. These approaches target the modulation of activity in the brain and, in turn, stimulate neural circuitry in regions affected by stroke (Fig. 1) [6–8]. For instance, invasive vagus nerve stimulation paired with rehabilitation has been shown to significantly improve arm recovery in chronic stroke [9]. Non-invasive brain stimulation (NIBS) may be more tolerable and acceptable to patients [10]. Repetitive transcranial magnetic stimulation (rTMS) is a non-invasive technique that uses magnetic fields to stimulate specific areas of the brain, which in turn induce electrical currents within targeted regions [11]. High-frequency rTMS exhibits excitatory effects and is typically targeted towards the affected (ipsilesional) motor cortex, while low-frequency rTMS, which exhibits inhibitory effects, is typically targeted at the unaffected (contralesional) motor cortex. Transcranial direct current stimulation (tDCS) is another non-invasive method of brain stimulation in which constant low electrical current is passed through electrodes strategically positioned on the scalp to modulate neuronal excitability [12]. Non-invasive or transcutaneous vagus nerve stimulation (tVNS) involves electrical stimulation of either the auricular or cervical branches of the vagus nerve through the skin [13].

Resting-state functional magnetic resonance imaging (rs-fMRI) can be used as a tool to understand the functional connectivity between brain regions responsible for motor activity post-stroke [14]. Previous research has noted the benefits of using resting-state connectivity measures versus task-based measures [15]. While task fMRI has provided novel insight into post-stroke plasticity, motor tasks in an MRI scanner are difficult due to various factors, including stroke-induced mirror movements and inconsistent task performance (such as variability in speed and amplitude of movement, degree of flexor synergy, and spasticity-related restrictions in range of motion). Rs-fMRI provides a stable and reliable measure of connectivity in the brain by investigating brain activities intrinsically without any task [14]. It offers a unique, coherent, and comprehensive method for assessing neural networks in people with a range of motor deficits. Resting-state fNIRS (rs-fNIRS) uses near-infrared light and can similarly assess neural connectivity in a task-free state, with greater temporal resolution than fMRI but lower spatial resolution [16, 17]. It offers an alternative in participants for whom MRI is contraindicated, and it can be used concurrently with multiple NIBS techniques to study the acute effects [18] whilst providing complementary mechanistic insights into the nature of post-stroke recovery.

The pivotal roles played by the ipsilesional and contralesional hemispheres in stroke recovery vary depending on the stage of recovery and the type of intervention used in rehabilitation [19]. The ipsilesional hemisphere, comprising the regions of interest associated with infarct location and thus containing vital areas for motor recovery, is often considered a primary target in rehabilitation strategies. It can be potentiated by high-frequency rTMS to increase its activity level, thus promoting the genesis of spared neural circuits likely dedicated to motor re-learning [19]. Conversely, the contralesional hemisphere frequently hyperactivates following stroke, partly due to loss of interhemispheric inhibition, and low-frequency rTMS is employed to inhibit that same area to prevent it from impairing ipsilesional recovery [19]. Several cross-sectional and longitudinal studies of arm function after stroke, monitoring both spontaneous and rehabilitation-mediated recovery have demonstrated that lower interhemispheric connectivity between homotopic brain regions, such as between both primary motor cortices, is correlated with increased stroke severity [14, 20]. Golestani et al. [21] demonstrated that early impaired connectivity can be a predictor of motor recovery. Furthermore, longitudinal rs-fMRI indicated that day 90 interhemispheric connectivity between ipsilesional and contralesional primary motor cortices remained reduced in patients with poorer recovery compared to those with better recovery. The additional effects of NIBS on rs-fMRI or rs-fNIRS are unclear; understanding these effects may provide insight into novel mechanisms of adaptive neural reorganization after stroke.

Here, we systematically review the evidence base for rs-fMRI and rs-fNIRS in NIBS for arm recovery following stroke and evaluate its correlation with clinical outcomes.

## Methods

### Search strategy

The systematic search strategy for studies that could potentially be included was conducted from the inception of databases until June 11, 2024, in EMBASE (1974 to June 11, 2024) and Medline (1946 to June 11, 2024) via the Ovid interface. Additionally, related terms and Medical Subject Headings (MeSH) terms were included to broaden the search scope and increase the likelihood of retrieving all pertinent studies. The full search strategy is outlined in Appendix 1. Other routes of study ascertainment included citation searches.

### Criteria for selection

We adopted the following inclusion criteria:

(i) **Article Type:** Clinical trials (randomized controlled trials).
(ii) **Population:** Adults (≥ 18 years old) with upper limb weakness after stroke (ischemic or hemorrhagic).
(iii) **Intervention:** NIBS, including TMS, tDCS, or tVNS.
(iv) **Outcome Measures:** (1) Clinical measures of arm function pre- and post-intervention, (2) rs-fMRI or rs-fNIRS pre- and post-intervention.
(v) **Language:** Study written in English.

We excluded articles focused solely on other post-stroke deficits, such as aphasia and balance, studies published as conference abstracts without a full article, case studies, and studies using task fMRI or fNIRS.

### Study screening

Two reviewers (WH and SSB) evaluated the titles and abstracts independently and read the full-text articles when necessary to determine whether the articles should be included or excluded. Any differing opinions were discussed by the reviewers, and consensus was sought by a third author (LS) if required.

### Data collection

Two reviewers (MA, SSB) extracted the following information from each included trial according to a standardized proforma: sample size, conditions, groupings, presence or absence of a control group, interventions and additional interventions, resting-state fMRI, clinical assessments, and outcome measures of resting-state MRI.

### Risk of bias assessment

The Cochrane RoB 2 tool was used to judge included randomized trials for risk of bias. It covers several domains of bias in terms of study design, conduct, and reporting. The studies were then judged as low risk, some concerns, or high risk across each of these areas. Particular issues of bias were identified and documented for each study. Risk of bias assessment was performed by two reviewers (MA and SSB), with differences discussed and consensus reached with the agreement of a third reviewer (LS) if required.

### Missing data

If data were not presented or missing from the full-text article, authors were contacted for clarification.

### Synthesis of data

Due to study heterogeneity in stimulation parameters, study interventions and methods of neuroimaging analysis, a meta-analysis was not performed. A qualitative synthesis of the evidence base was determined through consideration of NIBS technique and neuroimaging modality.

### PRISMA statement

The study was conducted in accordance with PRISMA 2020 guidance. A completed checklist is outlined in Appendix 2. The review was not previously registered.

## Results

### Study selection

**Fig. 2:**
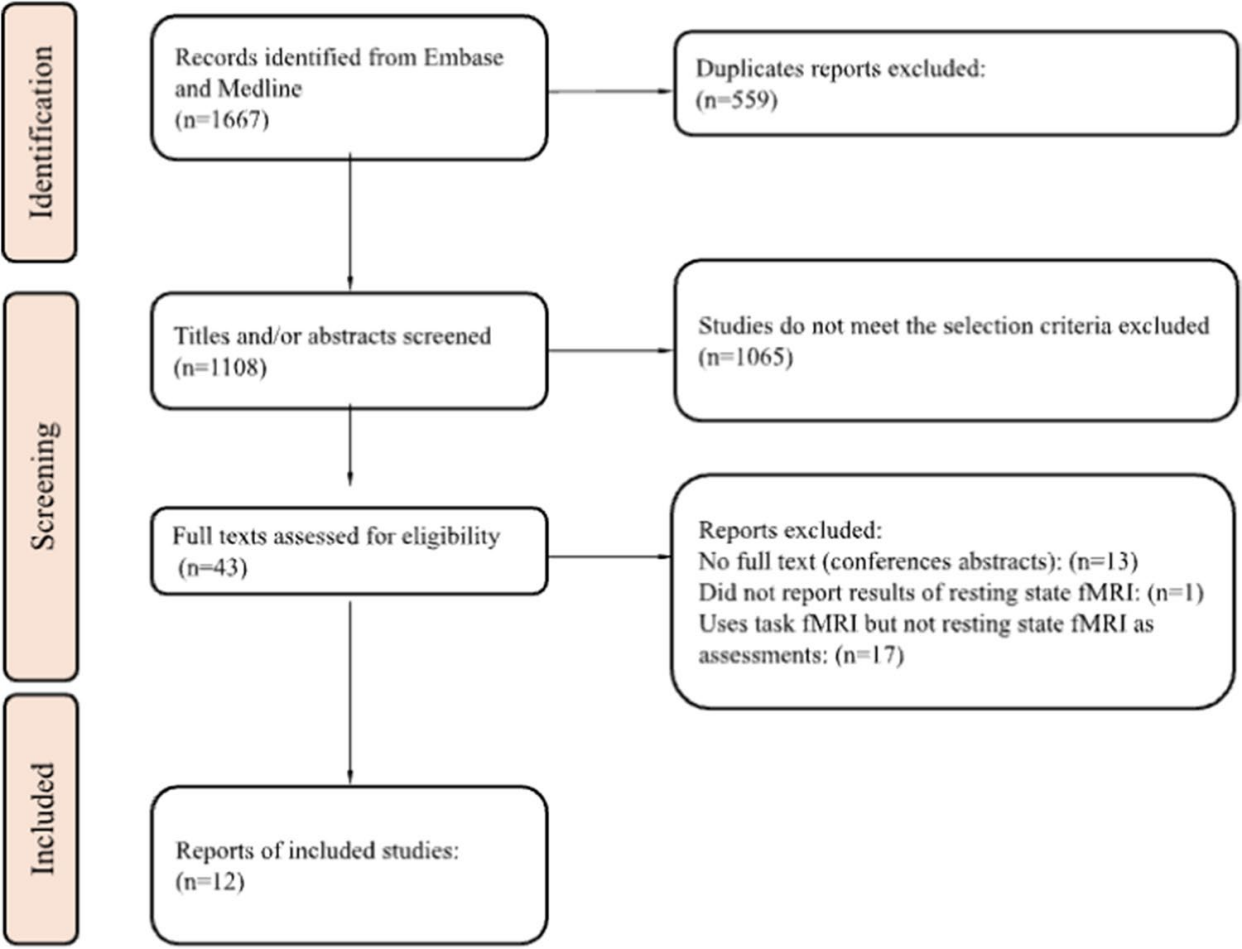
Study Selection

Our searches identified 1,667 titles and abstracts. After removal of duplicates, 1,115 unique references remained. The study selection is outlined in Fig. 2.

### Study characteristics

Twelve studies met the inclusion criteria. Baseline study cohort characteristics are outlined in Table 1. The total number of participants in this review study was 393. The combined age range of participants across studies was 30 to 86 years. Most studies evaluated rTMS in the subacute phase (< 1 month) following stroke.

**Table 1:** Baseline characteristic of study cohorts in included studies. *Participants with stroke and resting state fMRI at 2 timepoints

Study Name	Country of Study	N *	Treatment Groups (N)	Age (Mean)	%Male Participants	Time Post-Stroke	Stroke Types Included	%Left Sided lesions	Location of Lesion	Mean Baseline FMA-UE
**rTMS Studies**										
Li et al. (2016) [22]	China	12	rTMS (7) Sham (5)	55.3	83	< 7 days	Ischaemic	33	Subcortical MCA	42.4
Volz et al. (2016) [23]	Germany	17	TBS (7) Control (10)	62.8	82	< 14 days	Ischaemic	76	Cortical (frontal), internal capsule, subcortical white matter, and basal ganglia	N/A
Gottlieb et al. (2021) [24]	Germany	28	LF-rTMS (14) Sham (14)	63.0	43	> 14 days	Ischaemic and Haemorrhagic	29	Unilateral MCA territory	25.7
Guo et al. (2021) [25]	China	33	HF-rTMS (11) LF-rTMS (12) Sham (10)	64.5	45	< 7 days	Ischaemic	100	Subcortical	37.7
Qin et al. (2021) [26]	China	41	LF-rTMS (23) Sham (18)	59.9	61	< 1 month	Ischaemic	44	Basal ganglia and surrounding areas	27.4
Chen et al. (2022) [27]	China	63	LF-rTMS + HF-rTMS (16) Sham LF-rTMS + HF-rTMS (15) LF-rTMS + sham HF-rTMS (16) Sham LF-rTMS + Sham HF-rTMS (16)	57.4	65	< 14 days	Ischaemic	43	MCA territory	14.9
Du et al. (2021) [28]	China	46	HF-rTMS (15) LF-rTMS (17) Sham (14)	53.2	83	< 14 days	Ischaemic	57	Cortical and subcortical	29.1
Lv et al. (2023) [29]	China	36	20 session rTMS (18) Control (18)	57.6	58	14—28 days	Ischaemic	56	Supratentorial	**7.3**
Qin et al. (2023) [30]	China	49	HF rTMS and PMS (20) LF-rTMS (15) Control (14)	58.6	63	1—6 months	Ischaemic	35	MCA territory	25.4
**tDCS Studies**										
Hsu et al. (2023) [31]	Taiwan	27	tDCS (13) Sham (14)	59.2	56	14–28 days	Ischaemic	41	Subcortical and brainstem	30.8
Unger et al. (2023) [32]	USA	17	tDCS (7) Sham (10)	62.6	71	> 6 months	Ischaemic and Haemorrhagic	65	Lesions included internal capsule, basal ganglia, corona radiata, putamen, cortical, caudate nucleus, and thalamus	42.3
**Dual Mode Stimulation Studies**										
Lee et al. (2018) [33]	Korea	24	Dual-Mode (12) rTMS (12)	55.4	71	< 28 days	Ischaemic and Haemorrhagic	50	Cortical and subcortical	42.7

Nine studies assessed rTMS solely (in various forms, such as high-frequency and low-frequency) [22–30]. Two studies focused on all types of tDCS (bihemispheric, single hemispheric) [31, 32]. Lee et al. assessed the combination of tDCS and rTMS (dual-mode stimulation) [33]. There were no studies of tVNS that met the inclusion criteria. Various clinical outcome measures of arm function were assessed across studies, but the majority used the Fugl-Meyer Upper Extremity Assessment (FMA-UE) (n = 11). Six studies appear to report on the total FMA-UE score, including sensation and joint domains (range 0–126) [22, 24–26, 31, 33]. Five studies reported on the motor subscore (range 0–66) [27–30]. Volz et al. reported on hand function [23]. Resting-state fMRI (rs-fMRI) data were available for all studies. No studies used rs-fNIRS. The interval between intervention with NIBS and the MRI scan was reported for 5 out of 9 rTMS studies, neither of 2 tDCS studies and was reported for the single dual-mode stimulation study (Table 4). Only one study included serial behavioural and MRI assessments at multiple timepoints following intervention. [31]. A comprehensive overview of stroke lesion location, rTMS stimulation protocols, details of stimulation and concurrent interventions is provided in Appendix 3.

### Risk of bias

Table 2 summarizes the risk of bias within studies. Four of the twelve studies (33%) had a high risk of bias.

**Table 2:** Risk of bias assessment within studies

Author	Random Sequence Generation	Allocation Concealment	Blinding of Participants and Personnel	Blinding of Outcome Assessment	Incomplete Outcome Data	Selective Reporting	Other Bias	Overall Assessment
**rTMS**								
Li et al. (2016) [22]	Unclear	Unclear	Low risk	Unclear	Low risk	Unclear	Low risk	Low risk
Volz et al. (2016) [23]	Low risk	Unclear	Low risk	High risk	Low risk	Unclear	Low risk	High risk
Gottlieb et al. (2021) [24]	Low risk	Low risk	Low risk	Unclear	Low risk	Unclear	Low risk	Low risk
Guo et al. (2021) [25]	Unclear	Unclear	Low risk	Unclear	Low risk	Low risk	Low risk	Low risk
Qin et al. (2021) [26]	Unclear	Unclear	Low risk	Unclear	Low risk	Low risk	Low risk	Low risk
Chen et al. (2022) [27]	Low risk	Low risk	Low risk	Low risk	Low risk	Low risk	Low risk	Low risk
Du et al. (2021) [28]	Low risk	Low risk	Low risk	Low risk	Low risk	Low risk	Low risk	Low risk
Lv et al. (2023) [29]	Low risk	Unclear	High risk	Unclear	Low risk	Low risk	Low risk	High risk
Qin et al. (2023) [30]	Unclear	Unclear	High risk	Unclear	Low risk	Low risk	Low risk	High risk
**tDCS**								
Hsu et al. (2023) [31]	Low risk	Low risk	Low risk	Low risk	Low risk	Low risk	Low risk	Low risk
Unger et al. (2023) [32]	Low risk	Low risk	Low risk	Low risk	Low risk	Some concerns	Low risk	Low risk
**Dual Mode Stimulation**								
Lee et al. [33]	Unclear	Unclear	High risk	Unclear	Low risk	Low risk	Low risk	High risk

### Repetitive transcranial magnetic stimulation

**Fig. 3:**
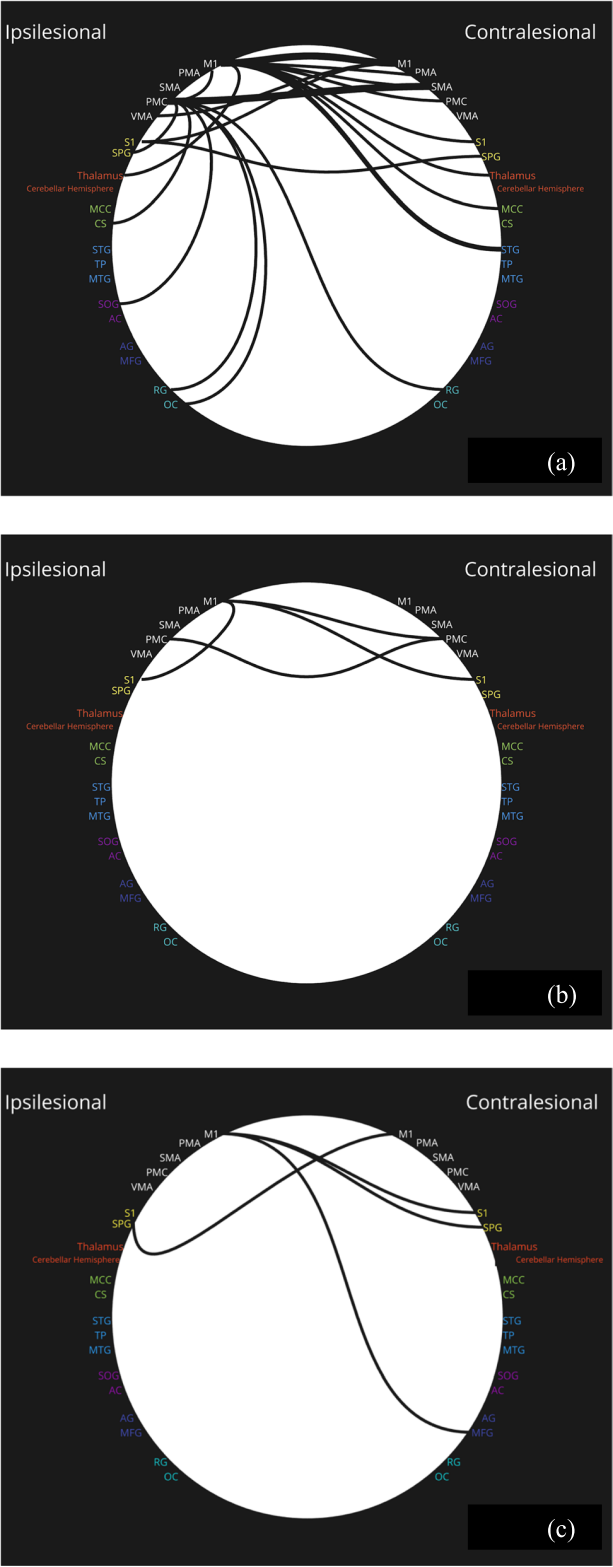
(**a**) – Areas of Increased Connectivity Following rTMS. Thicker lines represent a stronger connection, as more studies have identified this relationship. KEY: Motor areas – white, Sensory areas – yellow, Subcortical and modulatory areas – red, Cingulate areas – green, Temporal areas – blue, Occipital areas – violet, Cognitive and executive function areas – dark blue, Other limbic and olfactory areas – cyan. (**b**) – Areas of Decreased Connectivity following rTMS. KEY: Motor areas – white, Sensory areas – yellow, Subcortical and modulatory areas – red, Cingulate areas – green, Temporal areas – blue, Occipital areas – violet, Cognitive and executive function areas – dark blue, Other limbic and olfactory areas – cyan. (**c**) – Areas of Increased Connectivity following tDCS. KEY: Motor areas – white, Sensory areas – yellow, Subcortical and modulatory areas – red, Cingulate areas – green, Temporal areas – blue, Occipital areas – violet, Cognitive and executive function areas – dark blue, Other limbic and olfactory areas – cyan

The details of Acquisition, Preprocessing, and Analysis can be found in Table 3. The details of stimulation, clinical, and resting-state fMRI outcome measures are summarised in Table 4. Nine studies focused solely on rTMS [22–29], with Qin et al. combining rTMS with peripheral magnetic stimulation [30]. Eight studies were performed primarily on participants within the first month post-stroke, whereas two were performed in individuals more than one month post-stroke [26, 30]. Seven studies investigated rTMS of the contralesional M1 in at least one treatment arm, and five used ipsilesional M1 rTMS in at least one treatment arm. Seven studies compared against sham stimulation groups, whereas two had a control group without stimulation [29, 30]. The baseline FMA-UE across groups varied greatly (mean 7.1–44.8) Fig. 3.

**Table 3:** Details of MRI Acquisition, Preprocessing, and Analysis procedures

		**rTMS**									**tDCS**		**Dual Mode Stimulation**
	**Study**	**Li 2016** [22]	**Volz 2016** [23]	**Gottlieb 2021** [24]	**Guo 2021** [25]	**Qin 2021** [26]	**Chen 2022** [27]	**Du 2022** [28]	**Lv 2023** [29]	**Qin 2023** [30]	**Hsu 2023** [31]	**Unger 2023** [32]	**Lee 2018** [33]
**Acquisition**	**Model and field strength**	Siemens MAGNETOM Skyra 3 T	Siemens Trio 3 T​	Siemens Magnetom Avanto 1.5 T	GE Signa HDxt 1.5 T​	Siemens Tim Trio 3 T	Philips Ingenia 3.0 T	Discovery MR 750, GE Healthcare, 3.0 T	Discovery MR750 3.0 T	Siemens 3 T scanner	3.0-T GE Discovery 750 MRI scanner	Siemens Trio 3 T scanner with a 12-channel	Philips ACHIEVA 3 T MRI scanner
	**Duration of acquisition**	Not stated	7 min	8 min	4 min and 40 s	6 min​	8 min and 20 s	6 min and 50 s	8 min​	6 min	15 min	6 min 10 s	5 min
	**TE/TR**	30 ms/2510 ms	30 ms/2200 ms​	50 ms/3000 ms​	40 ms/2000 ms​	21 ms/2000 ms​	30 ms/2000 ms	30 ms/2000 ms	30 ms/2000 ms​	21 ms/2000 ms	30 ms/3000 ms	29 ms/2800 ms	35 ms/3000 ms
	**Eyes open/eyes closed**	Eyes closed	Eyes open​	Not stated	Eyes closed​	Eyes closed​	Eyes closed​	Eyes closed	Eyes closed	Not explicitly stated	Eyes open	Eyes closed	Eyes closed
**Preprocessing**	**Software and Toolbox**	DPARSF in SPM	SPM8	SPM12	SPM12	SPM12 & CONN toolbox​	DPARSF in SPM in MATLAB 9.2​	DPARSF in SPM in MATLAB	SPM12 and RESTPLUS V6.1	RESTplus, SPM12	SPM12, MATLAB 2018b, and in-house scripts	MATLAB, Analysis of Functional NeuroImages	SPM8, MATLAB R2014b
	**Lesion masking**	Not stated	Yes	Yes	Not stated	Yes	Yes	Yes	Yes	Yes	Not stated	Yes	Yes
	**Slice timing correction**	Yes	Not stated	Not stated	Yes	Yes	Yes	Yes	Yes	Yes	Yes	Yes	Yes
	**Realignment**	Yes	Yes	Yes	Yes	Yes	Yes	Yes	Yes	Yes	Yes	Yes	Yes
	**Coregistration**	Yes	Yes	Yes	Yes	Yes	Not stated	Yes	Yes	Yes	Yes	Yes	Yes
	**Normalisation**	To MNI space	To MNI	To MNI	To MNI	To MNI	To MNI	To MNI	To MNI	To MNI	To an Asian brain template	To MNI	To standard template space
	**Smoothing (full width at half maximum Gaussian Kernel)**	4 mm	8 mm	8 mm	8 mm	6 mm	8 mm	8 mm	6 mm	6 mm	6 mm	2.4 mm	6 mm
	**Band-pass filtering**	0.01—0.08 Hz	0.01—0.08 Hz​	0.008—0.12 Hz​	0.01—0.08 Hz	Low-pass at 0.15 Hz​	Not stated	Not stated	0.01—0.08 Hz​	0.01—0.08 Hz	0.01—0.1 Hz	0.01—0.08 Hz	0.009—0.08 Hz
**Analysis**	**Seed-based analysis**	Yes	Yes	Yes	Yes	No	Yes	Yes	Yes	No	Yes	Yes	Yes
	**Independent Component Analysis**	No	No	No	Yes	Yes	No	No	No	No	No	No	No
	**ALFF**	No	No	No	No	No	No	No	No	Yes	No	No	No
	**Graph Theory Approaches**	No	No	No	No	No	No	No	No	No	No	No	Yes

**Key**

ALFF - Amplitude of Low-Frequency Fluctuations

CONN - Connectivity Toolbox

DPARSF - Data Processing Assistant for Resting-State fMRI

MATLAB - MathWorks - Matrix Laboratory

MNI - Montreal Neurological Institute

SPM - Statistical Parametric Mapping

**Table 4:** Clinical and resting state fMRI outcome measures across studies

Author	Group	Study Duration	Time from end of intervention to MRI	Mean Baseline FMA-UE (SD)	Mean Post- Intervention FMA-UE (SD)	Mean Increase in FMA-UE	rs-fMRI Outcomes
Li et al. (2016) [22]	HF-rTMS	10 days	Not specified	40.6 (8.5)	51.6 (6.0)	11.0	Compared to Sham: Increased FC between ipsilesional M1 and contralesional M1, SMA, postcentral gyrus, superior temporal gyrus and bilateral thalamus. Decreased FC between ipsilesional M1, postcentral gyrus, middle frontal gyrus and superior parietal gyrus
	Sham	10 days		44.8 (7.6)	54.6 (4.3)	9.8	
Volz et al. (2016) [23]	iTBS	10 days	1 day	N/A	N/A	N/A	Compared to baseline: Increased FC between ipsilesional M1 and contralesional M1, contralesional dorsal PMC, ipsilesional MCC and bilateral SMA
	Sham	10 days		N/A	N/A	**N/A**	Compared to baseline: Decreased FC between ipsilesional M1 and contralesional motor areas
Gottlieb et al. (2021) [24]	LF-rTMS	12 days	3–4 days	27.7 (21.9)	34.1 (25.5)	6.4	Compared to baseline: Increased FC between ipsilesional M1 and the left angular gyrus
	Sham	12 days		23.7 (21.0)	27.2 (23.5)	3.5	No significant changes
Guo et al. (2021) [25]	HF-rTMS	10 days	Immediately	38.5 (22.6)	54.6 (19.8)	16.2	Compared to baseline: Increased FC in ipsilesional M1, SMA and premotor area Increased FC between ipsilesional M1, ipsilesional SMA and contralesional M1; contralesional SMA, ipsilesional SMA and contralesional premotor areas Compared to LF-rTMS: Higher FC between ipsilesional M1 and contralesional premotor area
	LF-rTMS	10 days		37.8 (15.1)	52.7 (20.0)	14.8	Compared to baseline: Increased FC in M1 and bilateral SMA
	Sham	10 days		36.7 (15.4)	40.6 (16.3)	3.9	
Qin et al. (2021) [26]	LF-rTMS	8 weeks	Not specified	26.3 (12.8)	49.1 (14.4)	22.9	Compared to baseline: Increased functional connectivity between sensorimotor network and visual network; frontoparietal network and default mode network.
	Sham	8 weeks		28.8 (12.0)	40.5 (10.9)	11.7	No significant changes
Chen et al. (2022) [27]	LF-rTMS + HF-rTMS (A)	4 weeks	1 day	16.3 (9.8)	50.0 (15.1)	33.7	Compared to Group D (double sham rTMS): Increased FC between ipsilesional postcentral gyrus and contralesional superior parietal gyrus; between contralesional precentral gyrus and postcentral gyrus. Decreased FC between contralesional postcentral gyrus and superior parietal gyrus Compared to Group B (sham LF-rTMS and active HF-rTMS): Increased FC between contralesional precentral gyrus and postcentral gyrus Compared to Group C (active LF-rTMS and sham HF-rTMS): Increased FC between contralesional postcentral gyrus and contralesional precentral gyrus, superior parietal gyrus
	Sham LF-rTMS + HF-rTMS (B)	4 weeks		18.7 (13.1)	33.7 (17.4)	15.0	Compared to Group D (double sham rTMS): Increased FC between contralesional precentral gyrus and postcentral gyrus. Decreased FC between contralesional precentral gyrus and superior parietal gyrus
	LF-rTMS + Sham HF-rTMS (C)	4 weeks		17.7 (10.2)	34.0 (16.8)	16.3	No significant differences compared to Group B or D
	Sham LF-rTMS + Sham HF-rTMS (D)	4 weeks		19.1 (14.5)	19.4 (14.7)	0.3	
Du et al. (202) [28]	HF-rTMS	5 days	< 24 h	33 (14)*	50 (10)*	17*	Compared to sham group: Increased FC between ipsilesional and contralesional M1; ipsilesional ventral premotor area and contralesional M1; ipsilesional ventral premotor area and SMA Compared to LF-rTMS: Increased FC between ipsilesional and contralesional M1
	LF-rTMS	5 days		29 (15)*	45 (10)*	16*	Compared to sham group: Increased FC between contralesional M1 and ipsilesional SMA
	Sham	5 days		25 (16)*	35 (5)*	10*	
Lv et al. (2023) [29]	LF-rTMS (20 session group)	6 weeks (rTMS for 4 weeks)	< 24 h	7.4 (3.6)	24.2 (6.7)	16.8	Compared with control group: Increased FC between ipsilesional M1 and ipsilesional precentral gyrus, postcentral gyrus, and cingulate sulcus plus contralesional temporal pole, middle temporal gyrus, and anterior cuneus Increased FC between ipsilesional premotor cortex and ipsilesional precentral gyrus, rectus gyrus, olfactory cortex, superior occipital gyrus, superior parietal gyrus, and cingulate sulcus plus contralesional supplementary motor area and rectus gyrus
	Control	6 weeks		7.1 (3.5)	17.5 (6.4)	10.4	
Qin et al. (2023) [30]	LF-rTMS	8 weeks	Not specified	25.3 ± 9.9	35.3 ± 7.4	9.9	Right Cerebellum: Shows improvement in motor control
	LF-rTMS and PMS	8 weeks		26.7 ± 8.9	41.9 ± 10.9	15.3	Compared to baseline: Increased ALFF in the contralesional supplementary motor area (SMA), cerebellum and middle frontal gyrus Reduced ALFF in contralesional postcentral gyrus Compared to LF-rTMS group: Increased ALFF in contralesional cerebellum. Reduced ALFF in ipsilesional precentral gyrus, middle frontal gyrus, inferior frontal gyrus and contralesional insula Compared to control group: Increased ALFF in contralesional cerebellum and medial frontal gyrus. Reduced ALFF ipsilesional precentral gyrus and supramarginal gyrus
	Control	8 weeks		23.6 ± 7.6	28.6 ± 7.0	4.9	Right Cerebellum: Less pronounced effect on motor control improvement compared to the combined group Right Medial Frontal Gyrus: Reflects enhanced activity in areas associated with motor task recognition
Hsu et al. (2023) [31]	Bihemispheric tDCS	2 weeks (duration of intervention)	Not specified	31.8 ± 17.5	45.4 ± 22.3	13.6	No group level differences over time at 2 weeks or 3 months At 2 weeks: Increased FC between ipsilesional M1 and bilateral S1 regions correlated with FMA-UE improvement
		3 month (follow-up)		31.8 ± 17.5	50.9 ± 19.3	19.1	No group level differences over time At 3 months: Increased FC between contralesional M1 to contralesional dorsolateral premotor cortex and ipsilesional ventrolateral premotor cortex to ipsilesional anterior intraparietal sulcus correlated with FMA-UE improvement
	Sham tDCS	2 weeks (duration of intervention)		30.6 ± 20.5	38.8 ± 22.1	8.2	At 2 weeks: Decreased FC between ipsilesional S1 and anterior intraparietal sulcus correlated with FMA-UE improvement
		3 month (follow-up)		30.6 ± 20.5	40.2 ± 24.1	9.6	At 3 months: Increased FC between ipsilesional M1 and ipsilesional dorsolateral premotor cortex correlated with FMA-UE improvement
Unger et al. (2023) [32]	Ipsilesional premotor cortex tDCS	5 weeks	Not specified	39.7 (14.2)	44.8 (NS)	5.1	Compared to sham tDCS: In those with moderate-severe baseline impairment, there was increased FC between the ipsilesional and contralesional dorsal premotor cortex Compared to baseline: Increased FC ipsilesional M1 and dorsal premotor cortex
	Sham	5 weeks		44.1 (12.0)	No significant changes	Not specified	Compared to baseline: Increased FC ipsilesional M1 and dorsal premotor cortex
Lee et al. (2018) [33]**	HF-rTMS and Contralesional M1 tDCS	2 weeks (intervention) 2 months (follow-up)	2 months	43.3 ± 19.5	71.8 ± 26.1	28.5	Compared to rTMS alone: No significant change in intrahemispheric FC from ipsilesional or contralesional M1 or interhemispheric FC of ipsilesional M1. Significant increase in interhemispheric FC from contralesional M1. Overall increased interhemispheric FC
	HF-rTMS	2 weeks (intervention) 2 months (follow-up)		42.0 ± 16.9	60.0 ± 23.6	18.0	

Note: Some studies used only the motor component of the Fugl-Meyer score (range 0–66), while others used the total score (range 0–126)

*Du et al. did not provide exact figures, so estimations were made based on their graphs. We have attempted to contact the authors for the actual data

** Whilst most studies used only the motor component of the Fugl-Meyer score (range 0–66), some have likely used the total score including sensation, range of motion and pain scores (range 0–126)

### Clinical outcomes

Eight of nine studies reported FMA-UE scores as outcome measures. All eight of these studies showed a significant increase in FMA-UE scores compared to baseline following intervention with rTMS. Seven studies (all but Li et al.) demonstrated a greater increase in FMA-UE scores with active rTMS compared to sham or control groups [22]. One study did not report FMA-UE but assessed grip strength. In this study, Volz et al. found that five days of intermittent theta burst stimulation delivered to the ipsilesional M1, prior to physiotherapy, resulted in greater relative hand grip strength (affected arm/unaffected) compared to sham stimulation [23].

In one of the three studies comparing ipsilesional (HF-rTMS) vs. contralesional (LF-rTMS) [25, 27, 28], Guo et al. reported that HF-rTMS resulted in a slightly larger increase in FMA-UE compared to LF-rTMS and sham rTMS [25], whereas the others showed no significant difference. Chen et al. assessed 20 sessions across rTMS in subacute ischemic stroke with four treatment groups consisting of combinations of active and sham HF- and LF-rTMS [27]. FMA-UE increased in all groups following the four-week intervention but was significantly greater in all groups containing at least one active rTMS stimulation. Furthermore, the combination of HF- and LF-rTMS resulted in significantly greater increases in FMA-UE than either modality alone or sham rTMS. These group differences persisted at three months.

In the single study of participants more than one month post-stroke, Qin et al. found that both LF-rTMS and LF-rTMS combined with peripheral magnetic stimulation (LF-rTMS + PMS) resulted in significant increases in FMA-UE compared to sham, with the combined stimulation showing a significantly greater increase in FMA-UE compared to LF-rTMS alone [30].

### rs-fMRI outcomes

All the studies showed that active rTMS resulted in changes in functional connectivity (FC) between bihemispheric sensorimotor regions. In the studies of HF-rTMS, interhemispheric connectivity was higher between the ipsilesional and contralesional primary motor cortices (M1) and between ipsilesional M1 and the supplementary motor area (SMA) [22, 25, 28]. Likewise, the studies of LF-rTMS exhibited significant increases in FC between the contralesional M1 and the ipsilesional SMA [28]. Post-treatment analyses further showed increased connectivity in the motor network (SMA and cerebellum) for patients treated with rTMS [28]. In comparison, the sham rTMS or control groups evidenced a modest decrease [23, 30] or no changes in functional connectivity [22–28]. Du et al. reported a different pattern of rs-fMRI changes with HF vs. LF-rTMS compared to sham stimulation and a significant increase in M1–M1 connectivity in the HF-rTMS group compared to LF-rTMS [28].

Three studies reported correlations between changes in clinical scores and changes in functional connectivity [25–27]. Of these, all showed a significant correlation. Du et al. showed that motor recovery correlated with increased FC in different regions in HF vs. LF-rTMS groups, which corresponded to the site of stimulation [28]. For HF-rTMS, FC with ipsilesional M1 and ipsilesional SMA, and contralesional M1 correlated with FMA-UE increases; for LF-rTMS, the positive correlations were associated with increased FC between contralesional M1 and ipsilesional SMA [28]. Chen et al. found a significant association between the change in FMA-UE scores after combined HF- and LF-rTMS; this effect was not present in other treatment groups containing either modality alone [27]. The functional connectivity changes seen here were: increased FC between contralesional precentral and postcentral gyri; increased FC between ipsilesional postcentral gyrus and contralesional superior parietal gyrus; decreased FC between contralesional postcentral gyrus and superior parietal gyrus. Furthermore, increases in FC between ipsilesional–contralesional precentral gyri and contralesional precentral and postcentral gyri were significantly correlated with FMA-UE improvement in this group.

We can also deduce from the studies the efficacy of sessions and discuss the potential of working out the optimal number of sessions required to notice improvement. For example, one study examining the optimal number of stimulation sessions revealed that participants who underwent 20 sessions of rTMS saw their Fugl-Meyer scores rise from 7.4 ± 3.6 to 24.2 ± 6.7, and those with 30 sessions improved from 7.6 ± 2.7 to 26.4 ± 6.8. These examples demonstrate that rTMS not only enhances motor function, but that the extent of improvement is closely related to the frequency and duration of the treatment. This suggests that determining the optimal number of sessions is crucial for maximizing benefits.

### Transcranial direct current stimulation

#### Clinical outcomes

There were two studies of tDCS alone. One study demonstrated no significant increase in FMA-UE following a 5-week intervention with premotor cortex tDCS prior to physiotherapy compared to sham tDCS [32]. In contrast, Hsu et al. used bihemispheric tDCS in subacute stroke patients and showed a significant improvement in mean (SD) FMA-UE from 31.8 (17.5) to 45.4 (22.3) compared to sham tDCS, which improved from 30.6 (20.5) to 38.8 (22.1) [31]. The latter study involved a much more intensive protocol of twice-daily sessions of tDCS lasting 20 min prior to 90 min of physiotherapy over a 10-day period. Furthermore, this was the only study to assess at a further post-intervention time point (3 months) demonstrating sustained improvements in the FMA-UE in the active group. There were no group level differences in functional connectivity between the active and sham group at either timepoint.

### rs-fMRI outcomes

While Unger et al. did not demonstrate significant clinical improvements at the group level, changes in FC were seen in a subgroup [32]. Increased FC between the ipsilesional and contralesional dorsal premotor cortex was observed in those with moderate-severe arm weakness at baseline. Across active and sham groups, there were increases in FC between ipsilesional M1 and the dorsal premotor cortex, with no group interaction, suggesting these relate to the effects of time and rehabilitation rather than tDCS. These latter changes correlated with reductions in proximal motor impairment in those with mild motor deficits.

After bihemispheric tDCS, while no intergroup differences over time surpassed the corrected threshold—this may be expected given the small sample size (only 19 having serial fMRI)—the authors did demonstrate increases in FC between ipsilesional M1 and bilateral S1 regions correlated with FMA-UE improvement in active tDCS [31]. In sham tDCS, the connectivity changes correlated with motor changes were restricted to the ipsilesional hemisphere, suggesting that bihemispheric tDCS may influence interhemispheric connectivity.

### Dual-mode stimulation

One study combined HF-rTMS with contralesional M1 tDCS versus HF-rTMS alone following a 2 week intervention with follow-up clinical and rs-fMRI assessment at 2 months post-intervention. Although there was an 11 point higher average improvement in the FMA-UE in the dual-mode group vs HF-rTMS alone, this was not statistically significant [33]. The rs-fMRI results indicated that the addition of cathodal tDCS to the contralesional M1 led to a significant increase in functional connectivity between the contralesional M1 and ipsilesional motor regions.

## Discussion

The current study systematically reviews the effects of rTMS and tDCS on motor recovery of arm function after stroke using clinical measures and resting-state fMRI. Both rTMS and tDCS are associated with improvements in clinical measures of arm strength in populations with subacute and chronic stroke. The rs-fMRI results indicate that the mechanism through which arm function improves with these treatment modalities is through increased connectivity in intra-hemispheric and interhemispheric sensorimotor regions.

Previously, several studies have provided an understanding of the roles of resting-state fMRI in spontaneous and rehabilitation recovery following a stroke. Notably, increased functional connectivity between the bilateral primary motor cortices (M1-M1) has been associated with significantly improved functional and motor outcomes in stroke survivors, especially after enhanced rehabilitation measures such as robot-assisted bilateral arm rehabilitation [34, 35]. Likewise, higher functional connectivity with ipsilesional frontal and parietal cortices, bilateral thalamus, and cerebellum has been noted in stroke patients with successful motor recovery, highlighting the value of these regions during recovery [20]. The correlation between the recovery of sensorimotor function and the restoration of interhemispheric functional connectivity in the sensorimotor network emphasizes the importance of functional connectivity as a marker for stroke recovery [36].

A large systematic review and meta-analysis by Hofmeijer et al. indicates that rTMS has a significant effect on improving the FMA-UE in individuals with stroke [37]. Understanding the mechanisms of recovery is an essential part of optimizing rehabilitation strategies. The current study highlights patterns of connectivity that arise from rTMS, which are associated with greater recovery of arm function. These include intra-hemispheric connectivity between the primary motor cortex (M1), premotor cortex, and supplementary motor areas, as well as improvements in interhemispheric connectivity, particularly between ipsilesional and contralesional M1.

This is complemented by studies using other modalities to assess cerebral function, such as task fMRI and TMS. For instance, LF-rTMS to the contralesional hemisphere has been shown to lead to more ipsilesional activation during motor tasks (e.g., potentially reducing interhemispheric inhibition) [38]. Zanona et al. found that ipsilesional S1 rTMS decreases interhemispheric asymmetry of the somatosensory cortex when combined with sensory stimulation [39].

A systematic investigation of rTMS by Chen et al. delineates the effects of LF-rTMS vs. HF-rTMS with four treatment groups consisting of combinations of active and sham conditions [27]. This study suggests that combined rTMS results in larger clinical improvements and greater connectivity between contralesional precentral and postcentral gyri than either modality alone [27]. Similarly, the addition of tDCS to HF-rTMS appears to confer a significant increase in interhemispheric connectivity from the contralesional M1 [33]. These findings support the additional benefits of combining stimulation modalities.

The number of tDCS studies was small in comparison to rTMS. A previous meta-analysis suggests a moderate effect size of tDCS combined with rehabilitation for stroke [40]. One of the studies did not show any clinical improvements in FMA-UE from bihemispheric tDCS compared to sham, but it did show that connectivity changes were associated with clinical improvements [31]. Previous studies have suggested large inter-individual variability in response to tDCS [41]; as such, identifying biomarkers of treatment response may help early stratification of potential responders versus non-responders in clinical practice.

Previous research has agreed with our findings regarding the role tDCS plays in potentiating functional connectivity to aid stroke motor recovery. In 2012, Sehm et al. demonstrated that the application of bilateral transcranial direct current stimulation (tDCS) on the primary sensorimotor cortices led to more pronounced enhancements in motor performance compared to unilateral tDCS. This effect was accompanied by alterations in resting-state functional connectivity across various brain regions [42]. In terms of enhancing functional connectivity, this was demonstrated by Alemanno et al., who showed that right anodal tDCS in chronic non-fluent aphasia patients enhanced interhemispheric functional connectivity [43]. While not directly related to motor improvement, some shared mechanisms may underlie post-stroke recovery of motor and language functions.

When comparing both TMS and tDCS modalities, it is evident that the focus of the stimulation is to modulate cortical excitability and functional connectivity to improve motor outcomes following a stroke. However, the studies suggest they achieve this through differing mechanisms. For example TMS uses electromagnetic induction to deliver focal, pulsatile stimulation, while tDCS passes a low-intensity direct current across broader regions of the cortex. 

Across the studies reviewed, high-frequency rTMS, typically above 5 Hz, facilitated the ipsilesional motor cortex. Low-frequency rTMS, usually at 1 Hz, inhibited the contralesional hemisphere, and both strategies helped rebalance interhemispheric excitability and enhance motor network reorganization for improved recovery [20, 23]. On the other hand, tDCS, whether applied bihemispherically with anodal stimulation over the ipsilesional side and cathodal stimulation over the contralesional side, or to a single hemisphere, demonstrated similarly beneficial effects on plasticity and motor function, although in a less focal manner [29].

It is worth recognising that Lee et al. studied a dual-mode protocol in which they combined high-frequency rTMS with contralesional M1 cathodal tDCS. They found the targeted effect of rTMS combined with the wider modulation of tDCS may have additive or synergistic advantages for stroke recovery, according to their observations of improved interhemispheric connection [31].

Interestingly, in this study, the intervention lasted 2 weeks but the post-intervention follow-up was at 2 months post-stimulation. Therefore, the changes in functional connectivity seen were not transitory related to the acute effects of stimulation.

Cassidy et al. [44] have previously repurposed the Bradford-Hill criteria when attempting to determine causality between functional connectivity and stroke recovery. This framework considers strength of association, consistency, specificity, temporality, biological gradient, plausibility, coherence, experiment and analogy. The current review demonstrates a degree of consistency with several studies showing relationships between interhemispheric connectivity (particularly between ipsilesional and contralesional M1) and clinical improvements. Furthermore, there is coherence between the existing evidence base of the role of ipsilesional and contralesional M1 activity obtained from studies of task fMRI. However, the changes in rsFMRI connectivity are not specific to NIBS with several studies demonstrating similar changes in connectivity in the sham stimulation cohorts. Additionally, the temporality of the effect has not adequately been identified i.e. it is not known whether changes in functional connectivity from NIBS precede or drive clinical improvement. As such, whilst changes in functional connectivity between interhemispheric sensorimotor regions are promising as candidate biomarkers for NIBS, further research is required.

## Limitations

There are several limitations to the current study. First, only studies written in English were selected. Second, comparison between studies is made challenging by varying fMRI acquisition and analysis pipelines across studies. Third, most studies have small sample sizes, which limits their statistical power. Fourth, while changes in functional connectivity and improvements in clinical outcomes from NIBS are correlated, further work is required to determine whether this is a causal relationship [44]. Fifth, some studies did not have sham stimulation as a control condition, leading to potential issues of bias. Sixth, while the baseline stroke severity (baseline FMA-UE) covers a wide range, there is a relative paucity of studies on individuals with severe stroke where the effects of the contralesional M1 may be supportive rather than inhibitory. Seventh, most studies were in subacute stroke, where spontaneous recovery is more likely and a potential confounder. Eighth, none of the rTMS studies incorporated serial rs-MRI to assess the consistency and temporality of the findings in the longer term. Ninth, several studies performed rs-fMRI within one day of the intervention making it difficult to determine whether the changes in connectivity were transient.

## Conclusion

The current review reveals changes in intra-hemispheric and interhemispheric connectivity in sensorimotor regions post-NIBS. These provide insight into the mechanisms of motor recovery from NIBS and support the continued investigation of combined neurostimulation approaches in stroke recovery.

Several future research recommendations can be made to further advance the understanding and application of TMS and tDCS in post-stroke recovery and determine whether rs-fMRI can serve as a biomarker. These include larger sample sizes, novel combinations of different neurostimulation modalities, the use of diverse populations (e.g., increased female participation), studies of other NIBS such as tVNS, and longitudinal studies with multiple post-intervention timepoints to assess the causal relationship between changes in functional connectivity and motor recovery and to determine whether the effects are transient, sustained or progressive.

## Figures and Tables

**Table 6:**
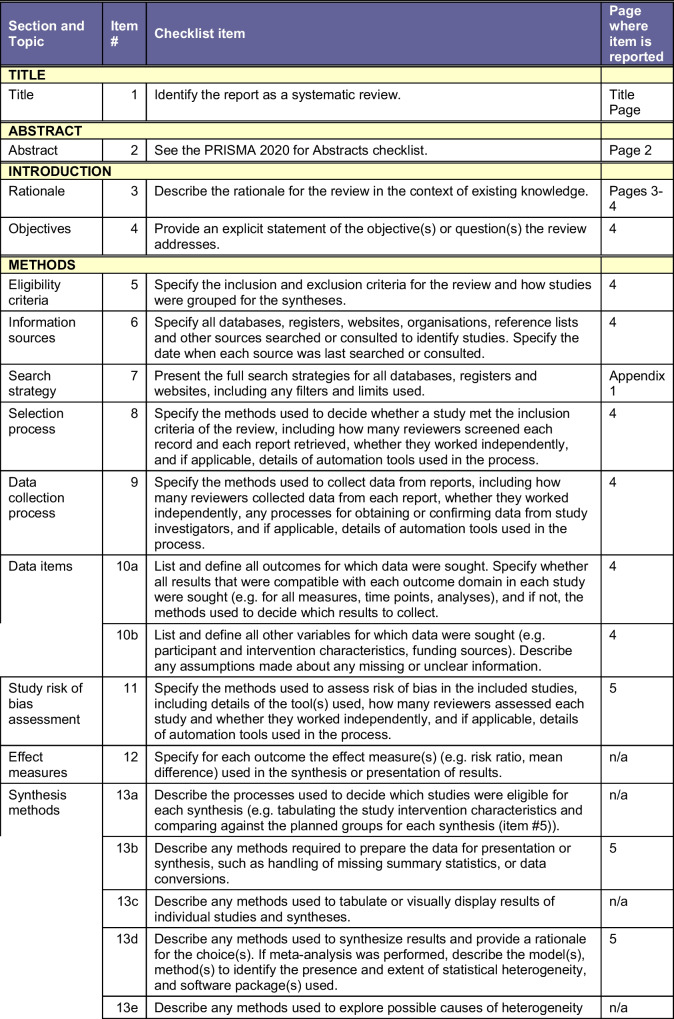

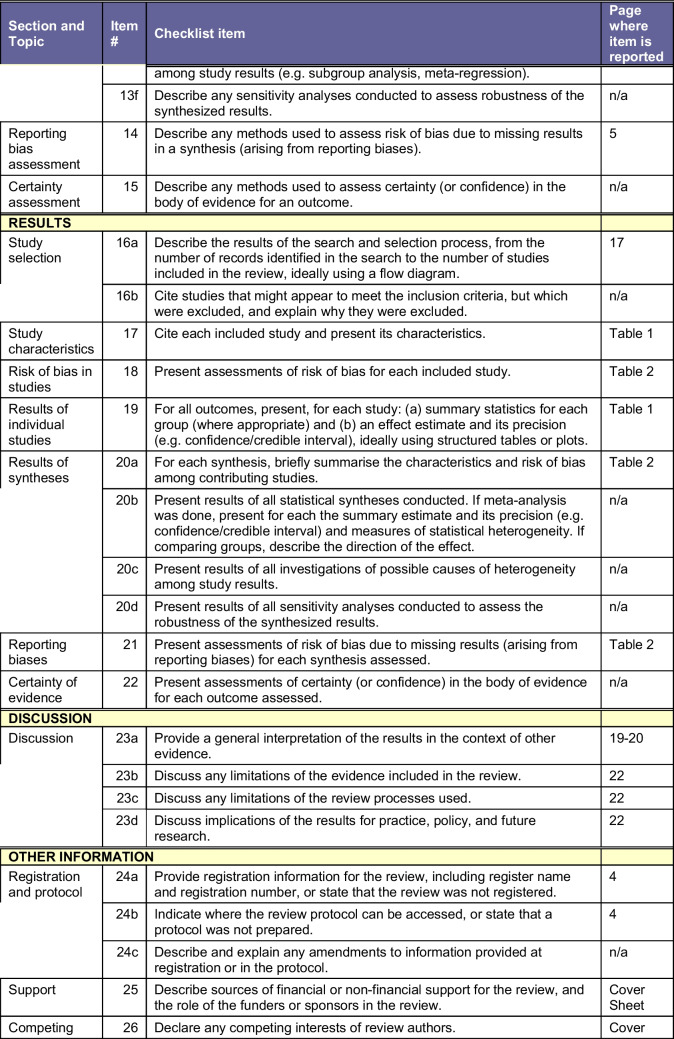

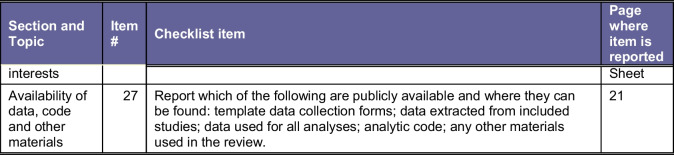
PRISMA 2020 Checklist

## Data Availability

The template data collection forms, data extracted and used for analysis are available to access via the corresponding author upon reasonable request.

## Appendix 1

Table 5

**Table 5:** Search strategy in EMBASE and MEDLINE

No.	Keywords	Results
1	(functional MRI or fMRI or MRI or Magnetic Resonance Imaging or BOLD or rsfMRI or connectivity). ab,ti.	1438953
2	(fNIRS or NIRS or near-infrared spectroscopy or Diffuse Optical Tomography or DOT or HD-DOT). ab,ti.	125735
3	(TMS or Transcranial Magnetic Stimulation or rTMS or TBS or Theta Burst Stimulation). ab,ti.	74972
4	(DCS or Direct Current Stimulation or tDCS). ab,ti.	92740
5	(VNS or Vagus Nerve Stimulation or Vagal Nerve Stimulation or Vagal Stimulation. Vagus Stimulation or auricular or tVNS or taVNS). ab,ti.	43405
6	(Stroke or poststroke or post stroke or Cerebrovascular Accident or CVA or Cerebrovascular Accident or Infarc* or cerebral ischem* or cerebral ischaem* or brain ischem* or brain ischaem* or hemorrhag* or haemorrhag*). ab,ti.	2128163
7	1 or 2	1559275
8	3 or 4 or 5	206521
9	6 and 7 and 8 = (1 or 2) and (3 or 4 or 5) and 6	1667

## Appendix 2

Table 6

## Appendix 3

Table 7

**Table 7:** Stroke Lesion Location, Details of Stimulation and Concurrent Intervention

Studies	Stroke Lesion Location	Details of Stimulation	Concurrent Intervention
rTMS			
Li 2016 [22]	12 in Anterior circulation (subcortical only)	Site: Ipsilesional M1 Pulses:50 trains of 20 pulses Frequency: 5 Hz Intensity: 120% of RMT of unaffected extremity. Total sessions: 10 sessions over 10 daysSham rTMS: not further specified	NS
Volz 2016 [23]	19 Anterior circulation (subcortical only):11 (Internal Capsule), 5 (Internal capsule & subcortical White Matter), 3 (Internal Capsule & Basal Ganglia)5 Anterior circulation with cortical involvement): 4 (frontoparietal cortex + subcortical) + 1 (frontal cortex + WM)2 Posterior circulation (pons)	Site: Ipsilesional M1 Pulses: 600 pulses over 3.5 mins Frequency: 50 Hz Intensity: 70% RMT Total sessions: 5 sessions over daysAs above but current directed away from head	Physiotherapy
Gottlieb 2021 [24]	28 in Anterior circulation (not otherwise specified)	Site: Contralesional M1 Pulses: 1200 Frequency: 1 Hz Intensity: 100% RMT Total sessions: 10 sessions over 12 daysSham: As above but current directed away from head	Physiotherapy
Guo 2021 [25]	33 in Anterior circulation (subcortical only)	HF-rTMS :Site Ipsilesional M1 Pulses: 1500 Frequency: 10 Hz Intensity: 90% RMT LF-rTMS: Site: Contralesional M1 Pulses: 900 Frequency: 1 Hz Intensity: 90% RMT Total sessions: 10 sessions over 10 daysSham: As per LF-rTMS but current directed away from head	Physiotherapy
Qin 2021 [26]	41 in Anterior circulation (subcortical only)	Site: Contralesional M1 Pulses: 1200 Frequency: 1 Hz Intensity: 90% RMT Total sessions: 40 sessions over 8 weeks Sham- as per above but current directed away from head	Physiotherapy
Chen 2022 [27]	50 in Anterior circulation (subcortical only)13 in Anterior circulation with cortical involvement.	LF-rTMS: Site: Contralesional m1 Pulses: 600 Frequency: 1 Hz Intensity: 90%HF-rTMS: Site: Ipsilesional M1 Pulses: 600 Frequency: 10 Hz Intensity: 90% RMTTotal sessions: 20 sessions over 4 weeks. Sham component (for all groups) involves current directed away from head	Physiotherapy
Du 2021 [28]	2 in Anterior circulation with (cortical only)36 in Anterior circulation (subcortical only)8 in Anterior circulation with subcortical and cortical involvement.	HF-rTMS:Site: Ipsilesional m1 Pulses: 1200 Frequency: 10 Hz Intensity: 100% RMT LF-rTMS: Site: Contralesional M1 Pulses: 1200 Frequency: 1 Hz Intensity: 100% RMT Total sessions: 5 sessions over 5 days. Sham: As above but current directed away from head	Physiotherapy
Lv 2023 [29]	72 (location not specified)	LF-rTMS: Site: Contralesional M1 Pulses: 1200 Frequency: 1 Hz Intensity: 80% RMT Total sessions: 10, 20 or 30 sessions over up to 6 weeks. Control- no rTMS	Physiotherapy
Qin 2023 [30]	49 in Anterior circulation (not otherwise specified)	LF-rTMS: Site: Contralesional M1 Pulses: 1200 Frequency: 1 Hz Intensity: 90% RMT LF-rTMS and PMS: As per LF-rTMS plus PMS Site: Erb's point affected arm Pulses: 1200 Frequency: 10 Hz Intensity: Motion Threshold Total sessions: 40 sessions over 8 weeks. Control:No rTMS or PMS	Physiotherapy
tDCS			
Hsu 2023 [31]	21 in Anterior circulation (subcortical only)6 in Posterior circulation	Bihemispheric tDCS Anode: Ipsilesional M1 Cathode: Contralesional M1 Duration of Session: 20 mins Intensity: 2 mA Total sessions: 20 sessions over 2 weeks. Sham: As above but direct current ceased after 2 mins.	Physiotherapy
Unger 2023 [32]	17 in Anterior circulation (subcortical only)8 in Posterior circulation	Anode: Anode: Ipsilesional Premotor Cortex Cathode: Contralesional supraorbital area Duration of session: 20 minutesIntensity: 1 mA Total sessions: 15 sessions over 5 weeks. Sham: As above but current turned on and off transiently (30 - 60 secs)	Physiotherapy
Dual Mode Stimulation			
Lee 2018 [33]	24 (location not specified)	HF-rTMS and Contralesional M1 tDCS (DUAL)HF-rTMS: Site: Ipsilesional M1 Pulses: 1000 Frequency: 10 Hz Intensity: 90% RMT tDCS: Cathode: Contralesional M1 Anode: Supraorbital ipsilesional side Duration: 20 mins Intensity: 2 mA Total sessions: 10 sessions over 2 weeks. HF-rTMS: As above without additional tDCS	NS
